# Determination of TTT Diagrams of Ni-Al Binary Using Neural Networks

**DOI:** 10.3390/ma15248767

**Published:** 2022-12-08

**Authors:** Leonardo Hernández-Flores, Angel-Iván García-Moreno, Enrique Martínez-Franco, Guillermo Ronquillo-Lomelí, Jhon Alexander Villada-Villalobos

**Affiliations:** 1 Center for Engineering and Industrial Development (CIDESI), Av. Playa Pie de la Cuesta No. 702, Desarrollo San Pablo, Querétaro 76125, Mexico; 2 Dirección de Investigadores por México-CONACYT, Consejo Nacional de Ciencia y Tecnología (CONACYT), México City 03940, Mexico

**Keywords:** TTT diagrams, artificial neural networks, thermal treatment, additive manufacturing

## Abstract

The heat treatment of a metal is a set of heating and cooling cycles that a metal undergoes to change its microstructure and, therefore, its properties. Temperature–time–transformation (TTT) diagrams are an essential tool for interpreting the resulting microstructures after heat treatments. The present work describes a novel proposal to predict TTT diagrams of the $\gamma^{\prime }$ phase for the Ni-Al alloy using artificial neural networks (ANNs). The proposed methodology is composed of five stages: (1) database creation, (2) experimental design, (3) ANNs training, (4) ANNs validation, and (5) proposed models analysis. Two approaches were addressed, the first to predict only the nose point of the TTT diagrams and the second to predict the complete curve. Finally, the best models for each approach were merged to compose a more accurate hybrid model. The results show that the multilayer perceptron architecture is the most efficient and accurate compared to the simulated TTT diagrams. The prediction of the nose point and the complete curve showed an accuracy of 98.07% and 86.41%, respectively. The proposed final hybrid model achieves an accuracy of 96.59%.

## 1. Introduction

Large industries such as energy or aerospace, among others, often use metal alloys that have different properties from those of pure metals. Even small amounts of an element can considerably change the mechanical properties of an alloy such as toughness, resistance to fatigue or corrosion [1]. The mechanical properties of metals are defined by their microstructure, which can be modified by thermal, thermomechanical, or thermochemical treatments [2]. The growth kinetics of phases or precipitates is limited mainly by the diffusion of elements on solidification. In addition, it is described by phase diagrams, time–temperature–transformation (TTT) diagrams, and continuous cooling transformation (CCT) diagrams.

Due to their excellent mechanical strength at high and low temperatures and their outstanding resistance to oxidation and corrosion, nickel-based (Ni) alloys are widely used in very demanding applications such as turbine components. However, for non-ferrous alloys such as those that are nickel-based, there are few reports of their respective TTT diagrams. Their main phase is gamma (matrix); nevertheless, other phases such as the *gamma prime* ($\gamma^{\prime }$, $Ni_{3}\left[Al,Ti\right]$) are more important since they are responsible for conferring their outstanding mechanical properties. In precipitation-hardened nickel-based alloys, the TTT diagrams define the solubilizing temperatures and the aging times and temperatures that generate the reinforcing phases ($\gamma^{\prime }$, $\gamma^{\prime \prime }$) and avoid phases that deteriorate mechanical properties, such as delta or Laves [3]. The concentration of these reinforcing phases, as well as their size and location, depends on heat treatments designed based on the TTT diagrams.

There are different ways to obtain the TTT diagrams of an alloy: (i) by experimentation, (ii) by simulation, and (iii) by prediction. The experimental investigation and design of TTT and CCT diagrams are both costly and time-consuming [4]. There is presently still a lack of such diagrams even for well-known nickel-based alloys because the experimental determination of the TTT diagrams is a nontrivial proposition [5]. In addition, for alloys with several elements, the simulation can take hours or even days. This type of program uses specific information on the elements that compose the alloy (mobility, conductivity, diffusivity, etc.) which is contained in costly databases. Alternatively, it may be advantageous to predict the TTT diagram of a particular alloy using suitable modeling techniques, where the chemical compositions are set as the input parameters. Due to the presence of a large number of variables and complex relationships between them, an artificial neural network (ANN) is thought to be the only solution for approaching this model. Recently, some scientific groups have begun to develop predictive systems of TTT diagrams for various alloys.

In the early period of this millennium, ref. [4] published the first approach to an ANN for the simulation of time–temperature–transformation (TTT) diagrams for titanium alloys. A standard *backpropagation multilayer* (BP) network was implemented and trained using data from the published literature. The prediction of the $Ti_{6}$–$Al_{4}$–V curves was studied. One of the disadvantages of this work is that it only determines the nose point through the network; the rest of the curve is created through formulas. Ref. [6] extends their previous work by comparing the results with experimental data. The models are used to track and analyze the influence of different parameters such as alloy composition and processing conditions. In the same year, ref. [7] reports an ANN to simulate the nonlinear relationship between the *beta transus* ($\beta_{tr}$), the temperature of titanium alloys, and the alloy chemistry. The authors configured their model to receive as input the chemical concentration of the alloy elements, while the output of the model is the temperature $\beta_{tr}$. The results showed a good agreement with the experimental data. Estimation of the temperature of $\beta_{tr}$ by thermodynamic calculation was performed for comparison.

The work presented by [5] addresses the identification of optimal chemical composition to precipitate an ultrafine bainite microstructure, where TTT diagrams of different compositions have been predicted using the conjugate gradient algorithm to reduce the experimental tests required. In another work, TTT diagrams reported in the literature were used to predict the curves of other steels having similar alloy components using a support vector machine (SVM) model. The proposed methodology, reported in [8], can be used for the prediction of TTT curves for cold-work steels and the prediction of phases for different heat-treatment methods. Accuracy greater than 90% is reported. Recently, refs. [9,10] report a combination of algorithms, including *BP, random committee, random forest, and bagging*, for predicting TTT diagrams with relevant descriptors (alloying elements, austenitization temperature, and retention time). The database was composed of data reported in the literature.

The prediction of CCT diagrams using ML techniques is also reported. The prediction of CCT diagrams in synthetic welding heat-affected zones for *Ni-Cr-Mo* steels using descriptors of relevant material is presented in [11], including chemical compositions and cooling rate. The authors describe that the *random forest* (RF) technique was the one that showed the best performance when predicting with greater precision the starting temperature of the ferrite and bainite transition. In addition, the *k-nearest neighbors* (KNN) strategy facilitated the prediction of the start temperature of the martensite transformation, and the use of a *random committee* (RC) was used to predict hardness. Ref. [12] published molecular dynamics (MD) simulations. This study proposes a combined method of classical nucleation theory and MD simulations. The method is used to calculate the TTT diagrams and the critical cooling rates of bulk metallic glass alloys using two compositions $Cu_{50}Zr_{50}$ and $Cu_{20}Zr_{80}$. The proposed method reasonably predicts the critical cooling rate based on the calculated TTT. The authors do not report the exact accuracy of the model.

Numerical methods for the calculation of CCT diagrams for low- and medium-carbon steels are reported in [13]. Comparisons were made with a multilayer perceptron (MLP) neural network. The input data are the chemical composition and the austenitization temperature. The results of the calculations consist of the temperature of the beginning and the end of the transformation, the volume fraction of the structural components, and the hardness of the steel after heat treatment. The authors do not report the effectiveness of the studied model.

The analysis of the process of austenite decomposition during the cooling process of various steel grades is studied in [14]. The authors propose a network of the long short-term memory type for the analysis of the transition path of the cooling curve. Experimental values from CCT diagrams were used as training data for the neural network.

In [15,16], different ML models are compared to predict the nose point and the TTT diagram for pearlitic steels and galvanized dual-phase steels. To compare the proposed models, different metrics are used, e.g., the correlation coefficient (R2), the root mean square error, and the mean absolute percent error (MAPE). The studied ANN architectures are implemented in the MATLAB environment and configured with different activation functions (*hyperbolic tangent, ReLu, Sigmoidal, etc.*) and up to 48 epochs. The authors report that the SVM architecture and multilayer backpropagation have the best results in the prediction tasks.

A prediction of the nucleation lag time of iron and steelmaking melts solely from elemental composition and temperature was produced via deep neural networks by [17]. The authors use data available in the literature and claim their work as the first published instance of the prediction of nucleation lag time that does not require composition specific empirical data. The implemented deep neural network achieved an average absolute scaled error of 39.9%.

Table 1 summarizes the related works that have already been published. The table shows the technical characteristics that each work used to determine the TTT/CCT diagrams. It is important to mention that there are few published works in this regard. This may be due to the fact that, within the scientific community, the use of specialized software (such as *Thermo-Calc™*) has been taken as the standard for the study of phase precipitation. However, the use of techniques belonging to AI within the TTT area has begun to gain strength due to the multiple advantages it offers, which is observed with the increase in research groups in this new sub-field.

In this work, a methodology to determine TTT diagrams of the $\gamma^{\prime }$ phase for the Ni-Al alloy is presented, using a novel hybrid architecture of ANNs trained with reported and simulated data. The manuscript is organized as follows: in the next section, the related works are discussed; then, the proposed methodology is presented; and then, the results are discussed. Finally, the conclusions are presented.

## 2. Materials and Methods

**Figure 1:**
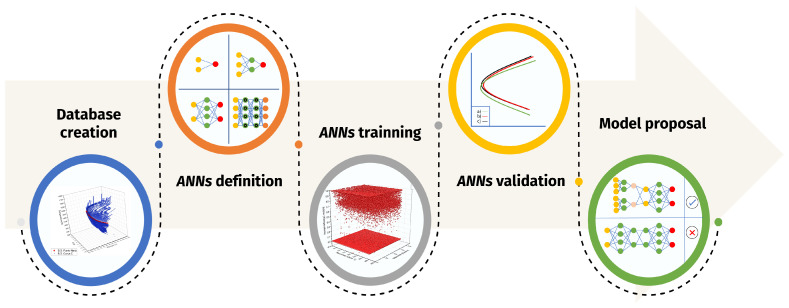
Proposed methodology for determining TTT diagrams using ANNs.

The present work implements a classical methodology for prediction tasks (see Figure 1). The first part addresses the creation of a database, which will be used in the training/validation tasks. Subsequently, the ML algorithms to study were selected, according to what is reported in the current literature. Next, the training and validation of each of the architectures were developed. This ends with the proposal of a model, in this case, a hybrid model, which showed better precision.

The current literature included a few works related to the use of intelligent systems to predict TTT diagrams of nickel-based alloys. The main reinforcing phase in nickel-based alloys is $\gamma^{\prime }$ [18]. This phase reinforces the matrix without reducing the fracture resistance of the material. In addition, it has a primitive cubic crystal structure (L12), with aluminum atoms at the corners of the cube and nickel atoms at the centers of the faces, according to [18]. Therefore, the present work will focus on the use of ANNs to predict the TTT diagram of the $\gamma^{\prime }$ phase in the NiAl alloy.

### 2.1. Database Creation

For any approach based on ML, it is necessary to define a database (DB) for the training and validation tasks. The current literature was reviewed to generate the DB. Few works have been reported by [19,20,21] addressing the calculation of TTT diagrams for NiAl binary. Due to these limitations, the DB was fed with some simulated diagrams using specialized software based on the CALPHAD methodology. There are several software packages on the market, for example, FactSage™, MTDATA™, PANDAT™, MatCalc™, JMatPro™, and *Thermo-Calc™*.

The *Thermo-Calc™* was used for the extensive calculations it can perform [22]. The Precipitation Module (TC-PRISMA) was also used, since it incorporates more functionality to *Thermo-Calc™* such as simultaneous nucleation, growth/dissolution, and coarsening under arbitrary heat-treatment conditions in multicomponent and multi-phase systems using Langer–Schwartz theory and the Kampmann–Wagner numerical approach. Further, two databases were used, the *TCNI8* and the *MOBNI5*.

**Figure 2:**
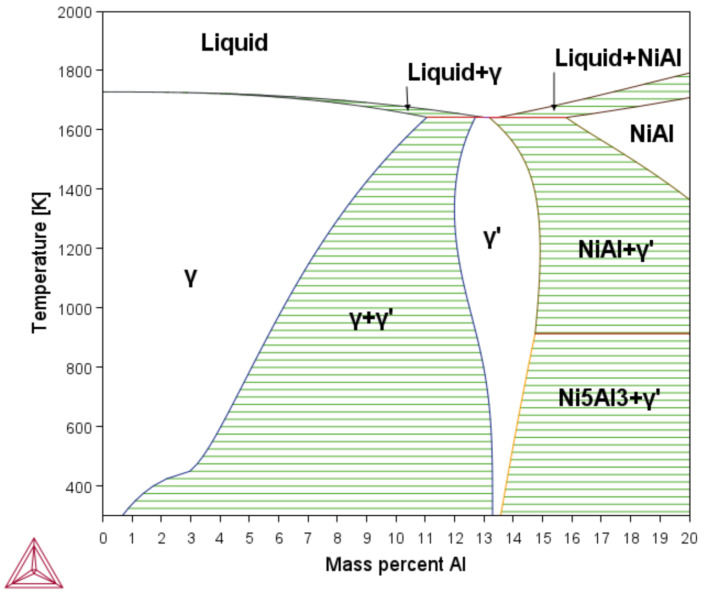
*NiAl* phase diagram in weight percent. Both diagrams simulated by *Thermo-Calc™*.

Using the phases diagram (see Figure 2), the minimum and maximum temperature ranges were determined, as well as the chemical composition in which the phases γ and $\gamma^{\prime }$ are present as stable phases and the mixing zone of these two phases. The diagram has a weight percentage range between 0 and 20. The ranges in the presence of a mixing zone of the phases γ and $\gamma^{\prime }$ were from 5.3 to 13.3 percentage weight of aluminum; from these limits, work began on the simulation of TTT diagrams through the *TC-PRIMA* module.

The *TCBIN* database was used to define the minimum and maximum percentages of Ni and Al in which the mixing zone was created, as well as the limits of the stable zone of the γ and $\gamma^{\prime }$ phases. As shown in Figure 2, the lower limit for the area of the mixing zone of the phases γ and $\gamma^{\prime }$ is at a $323.15{\;}^{\circ }$K, which ranges from 0.70% to 13.3% of aluminum percentage. The upper limit is at the temperature of $1640.15{\;}^{\circ }$K, which ranges from 11.0% to 12.7% of aluminum.

Once the range of chemical composition where the $\gamma^{\prime }$ phase precipitates were set, all the parameters to perform the simulation was defined. The parameters were the chemical composition, elastic properties, simulation temperature, phase to be precipitated, and grain size. Table 2 shows the values for each parameter. The simulations began with the lowest composition of aluminum (5.3% Al and 94.7% Ni), determining the first TTT of the phase $\gamma^{\prime }$, up to the maximum value of 13.3% Al with an increase by 0.1 % wt. Values outside this range cannot be simulated by *Thermo-Calc™*, due to the short precipitation times (microseconds or nanoseconds) and the limited presence of the $\gamma^{\prime }$ phase.

**Figure 3:**
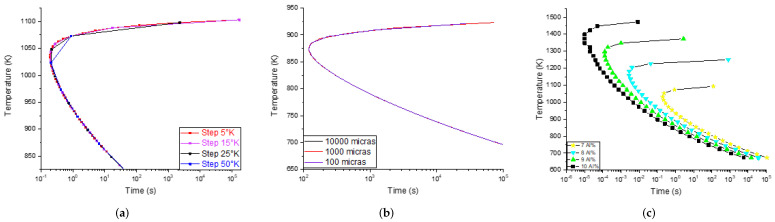
Comparison of simulated TTT diagrams varying (**a**) *steps*, (**b**) grain size and (**c**) chemical composition. It is observed that only the chemical composition has a considerable impact on the TTT diagrams. Due to this, the *steps* and grain size were discarded as inputs to the ANNs.

The grain size and step parameters do not have an influence on the precipitation times and temperatures of the phases (*Thermo-Calc™* calculations), as can be seen in Figure 3. Hence, these parameters were not taken into account for the simulation. As a result, a compilation of 96 TTT diagrams was obtained (simulated) to compose the DB; each diagram corresponds to a different chemical composition.

With all sets of simulated TTT diagrams, two databases were created. Each DB is composed of the Euclidean coordinates of the points that describe the curve. The first, and following the proposed by [4], was used to predict only the nose point of the diagrams. The nose point is the shortest time that a phase requires for its precipitation. The second DB was used to predict the complete TTT curves. In this case, the curves of each diagram are divided from the nose point, into an upper and lower part (sub-curves). In addition, since the TTT curves are not symmetrical, values for the shorter sub-curve were interpolated to obtain the same number of coordinates for both segments. Each DB was divided into two, the nose point DB consists of 96 data points, and the complete curve DB consists of 19,296 data points. The first part was used to train the models (66% of data) the second part was used to validate the models (33% of data).

### 2.2. ANN Definition

There are several ML architectures for the experimental design. We refer, in the first instance, to those that have already been reported. Support vector machines (SVM) [23] and multilayer perceptron (MLP) [24] have been used in the literature for TTT/CCT prediction. On the other hand, it is proposed to use an LSTM architecture because it is a type of architecture that is designed with a memory cell to preserve the state of the activation function for a long period [25]. It is a recurring network.

It is necessary to validate the performance of each architecture. There are several standardized metrics. In this work, two metrics were used to validate the performance of each ANN. On the one hand, the *Score* metric was evaluated, which indicates the degree of similarity that the predicting data has with respect to those it has for validation; closer to 1 indicates greater effectiveness. It was decided to use those architectures with a value greater than 0.95. On the other hand, the root mean square error (RMSE) was also used. This metric indicates the absolute fit of the model to the data, and how close the observed data points are to the model’s predicted values. Being a minimization metric, it is expected to have the smallest possible value. The RMSE is calculated as:

(1) $$RMSE=\sqrt{\frac{\sum_{i=1}^{N}{\left(Prediction_{i}-Current_{i}\right)}^{2}}{N}}$$

### 2.3. Experimental Design

The implemented experimental design contemplates four experiments (see Table 3). The predictions of the nose point and the complete curve were evaluated individually. From here on, each of the experiments is referred to as Exp. 1, Exp. 2, Exp. 3 and Exp. 4.

Exp. 1 was comprised of two strategies, the first has as input the chemical composition to predict the temperature. The second one is to predict the time, having as input the chemical composition. For Exp. 2, two strategies were also implemented, and data was shared between them. The first one has as input the chemical composition to predict the temperature. This value was an input along with the chemical composition for a second network to predict the time. Exp. 3 was comprised of only one ANN, which has the chemical composition as input, and the temperature and time parameters as output. Similarly, Exp. 4 has only one strategy; this network consists of two inputs (chemical composition and temperature) to obtain the time as output.

For Exp. 1, Exp. 2, Exp. 3, the precision of prediction was calculated according Equation (2). The precision was calculated as the ratio between the number of positive values (time–temperature pair) correctly predicted to the total number of values predicted (either correctly or incorrectly). A threshold of ±10 s was defined to consider the prediction of a value as true. The precision measures the model’s accuracy in predicting the time–temperature pairs as positive. When the model makes many incorrect positive predictions or few correct positive predictions, this increases the denominator and makes the precision small. On the other hand, the precision is high when the model makes many correct positive predictions (maximizing true positives) or when the model makes fewer incorrect positive predictions (minimizing false positives).

(2) $$Precision=\frac{Prediction_{true\_positive}}{Prediction_{true\_positive}+Prediction_{false\_positive}}$$

For Exp. 4, the precision was calculated using the Kolmogorov–Smirnov goodness of fit test (K-S test), which assesses how a dataset is significantly different from the probability model specified under the null hypothesis and fits to the same distribution. The K-S test statistic quantifies the distance between the measured dataset distribution and that of the observed dataset [26].

Different configuration parameters were modified for each analyzed algorithm (e.g., *kernel, gamma, solver, number of layers, activation function*). For the MLP and LSTM networks, different alternatives were implemented: single layer, bilayer, and trilayer. The idea is to obtain the best performance with the least number of layers and the least number of neurons. The LSTM/SVM/MLP algorithms were used for the prediction of the complete curve. Similarly, SVM/MLP algorithms are also used to predict the nose point.

## 3. Results

As previously mentioned, two databases were used. One was used to train an algorithm to predict the nose point and the other one to predict the complete curve. Subsequently, the different evaluated algorithms (SVM, MLP, and LSTM) were configured with a specific set of parameters for each of the four different experiments proposed in Table 3. The algorithms that obtained a higher *Score* and lower RSME during training were selected as viable options.

The prediction results were analyzed to validate the accuracy with which both the nose point and the entire curves were predicted. For this, different chemical compositions and different temperature ranges (within the maximum–minimum previously defined) were randomly evaluated. Since *Thermo-Calc™* is a reference within the scientific/industrial community to calculate the phases’ precipitation, this work assumes as a highly precise reference the TTT diagrams calculated by *Thermo-Calc™*. The predicted results are compared against to the diagrams simulated by *Thermo-Calc™*.

### 3.1. Nose Point Prediction

Table 4 shows the measured metrics (RMSE and *Score*) to validate the performance of the considered algorithms (SVM, MLP) to predict the nose point. (see the experimental design in Table 3). It is observed that to predict the time, Exp. 1 is the best option, using the MLP (three-layer) algorithm configuration, with the lowest RMSE (279.10 s), as well as a *Score* of 0.9999. On the other hand, the best configuration to predict the temperature was Exp. 3 with the MLP (mono-layer) algorithm, with an RMSE of 3.37 ${}^{\circ }$K and a *Score* of 0.9997. It is important to mention that the SVM algorithm has the worst results. This is probably due to the weakness of the soft-margin-optimization problem. This resulted in the hyperplanes being skewed to the minority class when imbalanced data was used in the training task. Another reason could be a wrong kernel selection; more suitable function kernels should be analyzed in future works.

Exp. 2 was the experiment that showed more deficiencies in the prediction of the nose point. This is because the output of the first network (strategy 1) was used as input to the second (strategy 2). There is error propagation in this procedure, which makes it highly sensitive to variations in input.

Therefore, the proposed model to predict the nose point is a dual network that includes the findings of Exp. 1 and Exp. 3 (see Table 3). Thus, this model will be composed of two neural networks. The first network will be a multilayer perceptron with three hidden layers (7-3-10 neurons) to predict the time. Configured with an activation function of *tanh* type and *Solver lbfgs*, with the chemical composition as input and the time as output. The second network is of the perceptron-multilayer-type composed of one hidden layer (6 neurons). Configured with an activation function of the type *tanh* and *Solver* of the type *lbfgs*, and as input the chemical composition and output the time and temperature. In this case, the time was discarded because it was already calculated with the previously described configuration. Then, the nose-point ordered pairs were formed with the predicted time of Exp. 1 and the predicted temperature of Exp. 3. This dual architecture optimizes performance and increases the accuracy of prediction results. The precision of this dual architecture was 98.07, using Equation (2).

Considering that the set of time values has a high cardinality (ranging from $1.4\times 10^{-7}$ to 168,000 s), a more complex algorithm (a larger number of hidden layers) is necessary for prediction; this is unlike the temperature set (ranging between 740 and 1590 ${}^{\circ }$K), which is simpler. This is the reason why this dual network optimizes the prediction of the nose point of TTT diagrams.

### 3.2. Complete Curve Prediction

A similar analysis to the one performed with Exp. 1/Exp. 3 was run with Exp. 4 to predict the complete curve. Different triples of values (Al%, $T_{max},T_{min}$) were randomly selected to predict the complete curve of the phase $\gamma^{\prime }$. The chemical compositions range between 5.3 and 13.3 Al. In a similar way, the maximum and minimum temperature ranges were taken with respect to those obtained from the Ni-Al binary diagrams in the *Thermo-Calc™* simulation. Subsequently, these triples were used as inputs for the three algorithms to be evaluated (SVM, MLP, LSTM). The MLP was configured for one/two/three layers, and LSTM was configured for one/two layers.

**Figure 4:**
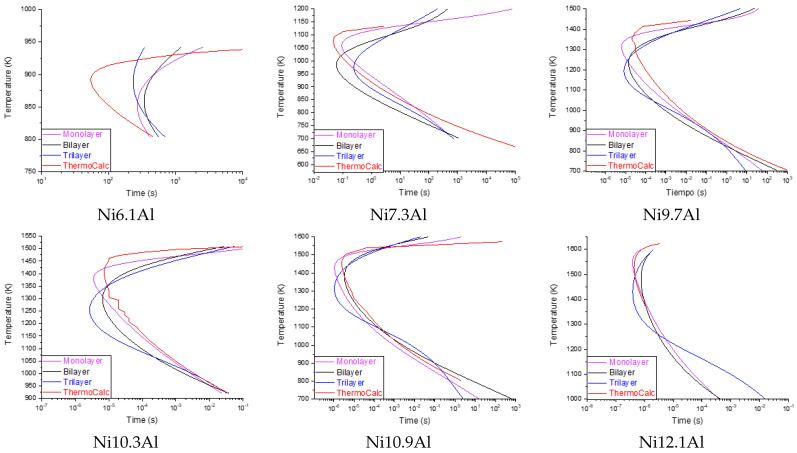
Complete curve prediction behavior of different MLP configurations against the results of *Thermo-Calc™* for different chemical compositions. It is observed that the MLP monolayer has the best performance.

Table 5 shows the RMSE values calculated for each algorithm evaluated for Exp. 4. It is observed that the values of the MLP and LSTM have a higher RMSE (with 1513.74 and 106,438.80 on average, respectively). In fact, the LSTM configured as a monolayer represented atypical results (outliers), so it was not possible to calculate the dispersion of the residual analysis. This atypical behavior of LSTM networks can be explained in relation to the amount of data that the algorithm requires to predict with acceptable precision. In addition, because there is little reported information (TTT diagrams) for the binary system studied here, it is not possible to develop a database of the necessary size. Due to these considerations, neither the LSTM nor SVM algorithms were considered for further analysis. On the other hand, it is observed that the MLP algorithm in the different configurations (1,2,3-layers) shows the best performance. Specifically, the MLP-monolayer has the best performance, with an average RMSE of 1457.40. It is also observed that as the chemical composition of Aluminum approaches the upper and lower limits, the RMSE tends to grow. This is because precipitation times are very fast (fractions of a second) or very slow (several hours), and the algorithm takes more runtime to converge to the minimum. Figure 4 shows the prediction performance of the MLP algorithm using the different configurations. The TTT curve calculated with ThermoCalc is shown within the same diagrams. As mentioned before, the MLP-monolayer is the best performer.

The accuracy achieved by each of the implemented algorithms to predict **Exp. 4** was also calculated. Table 6 shows the results of the Kolmogorov–Smirnov goodness-of-fit test [27]. It is observed that the MLP-monolayer has an accuracy higher than 80%, in some Al%, reaching 95%. The same phenomenon is observed as in the evaluation of the RMSE, at the limits of the percentage of aluminum, there is less precision in the prediction. The average precision to predict the complete curve is 86.41% for the MLP-monolayer model.

### 3.3. Hybrid Model Proposal

Based on the results obtained from the experimental design (Exp. 1/Exp. 2/Exp. 3/Exp. 4), the model for predicting the nose point has a precision of 98.07%. On the other hand, the best model to predict the complete curve has an average accuracy of 86.41% (MLP-monolayer), which translates into temperature differences greater than 329 °K at some points. From a practical point of view, this difference is unacceptable, since there is a risk of precipitating unwanted phases during heat treatment. Therefore, it is concluded that the results obtained from Exp. 4 do not have adequate precision.

Hence, a hybrid model is proposed which combines the previous best-ranked algorithms, with a better performance to predict time and temperature, both from the nose point and from the complete curve. The proposal is formed by a dual perceptron multilayer network which includes a trilayer network of Exp. 1 and a single layer of Exp. 3 to predict the nose point, as well as the single layer perceptron-multilayer network of (Exp. 4) to predict the upper and lower parts of the complete curve. The determination of the complete curve starts at the nose point.

To predict the complete curve, our proposal receives as input the percentage of aluminum, temperature limits (lower/upper) and the temperature step to perform the calculation. Since nickel is the balance element, it is enough to know the percentage of aluminum to define the chemical composition of the binary alloy. For this study, the temperature range of 600 to 1500 K was selected, taking into account that the maximum temperature for the calculation must be between the solvus and solidus temperatures, and the minimum temperature must be the one in which there is no precipitation of the phase under study.

**Figure 5:**
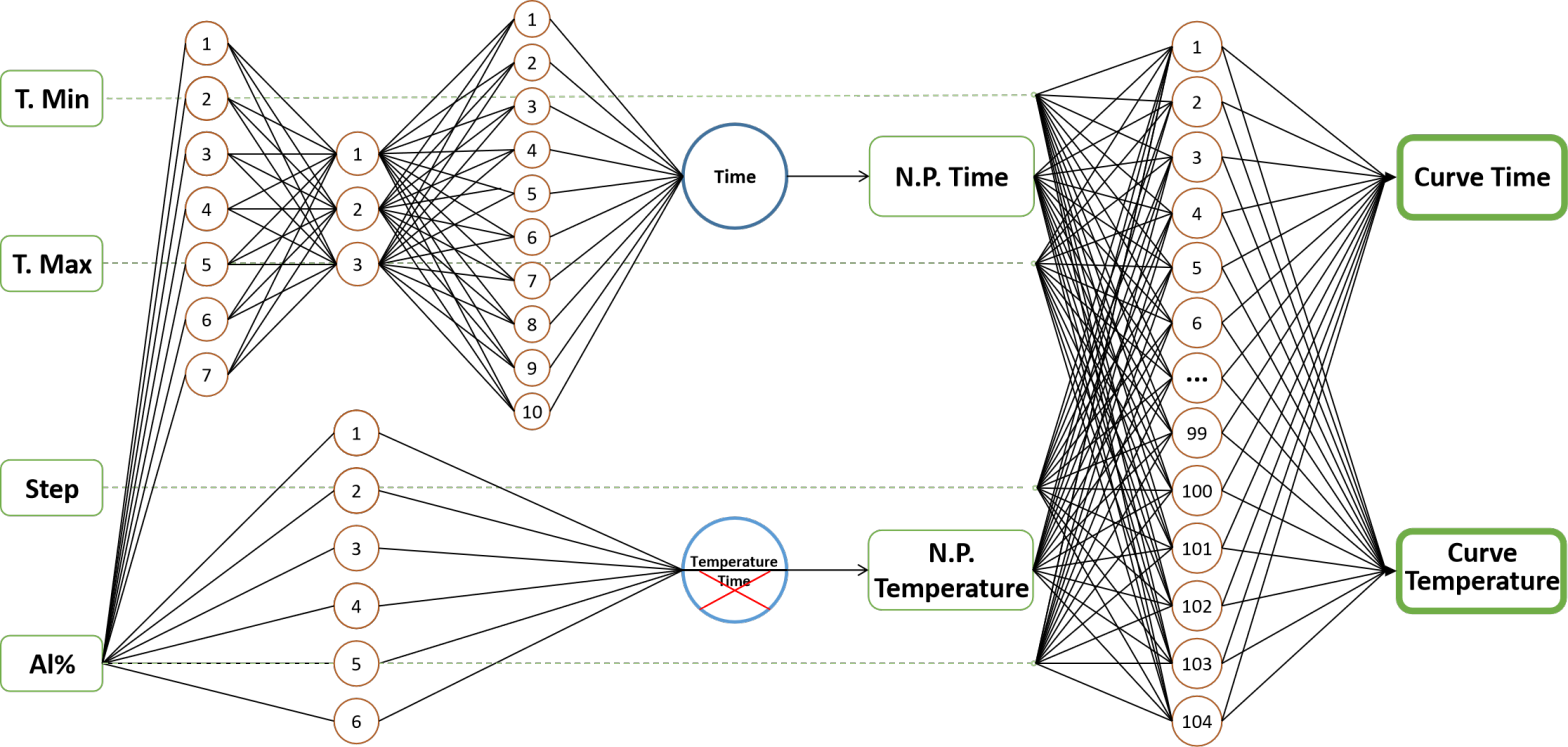
PredictionScheme of the proposed hybrid network. The model receives four inputs for the prediction of the nose point, the top and bottom of the TTT diagram.

Although our hybrid model has the competence to predict the entire diagram, it is only possible to predict within a specific temperature range. The upper-temperature limit is usually defined by the solvus temperature for the corresponding phase. The lower limit is the minimum temperature to obtain 1 percent of the phase. These values can be estimated according to the user’s expertise, or from experimental results or literature reports. The reason for this temperature setting is that, often, there are limitations derived from the type of oven that will be used in the heat treatment. The steps define the resolution of the curve: a small step implies a better resolution. Figure 5 illustrates the proposed final scheme, which it is made up of three different neural networks. The temperature/time values previously defined for the dual network in the nose-point prediction are used as input for the second network.

**Figure 6:**
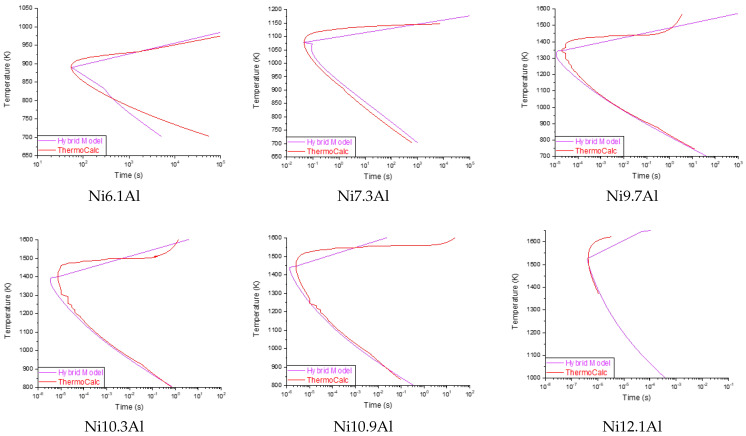
Prediction results for several chemical compositions of the proposed hybrid model against the *Thermo-Calc™*. Table 7 shows the precision for each plot. As the chemical composition approaches the lower/upper limits, the accuracy decreases.

The prediction results of the proposed hybrid model are presented in Figure 6. Improved precision is observed in the prediction of the curves compared to the results obtained from Exp. 4, as well as a smaller deviation with respect to the data simulated by *Thermo-Calc™*. Table 7 presents the precision and the RMSE of the diagrams presented in Figure 6. The prediction limits in the diagrams also affect the accuracy; as has already been discussed, these range from nanoseconds for chemical compositions with high Aluminum content due to the rapid precipitation of the γ’ phase, and, for the low amount aluminum, which occurs in terms of hours. Future works will address this phenomenon, seeking to optimize the prediction limits.

The overall accuracy of our proposal is **96.59**, measured along the lower/upper bounds of the workspace.

## 4. Conclusions

The present work proposes a strategy based on neural networks to predict, with high precision, temperature–time–transformation diagrams for the binary *NiAl*.

A database was built by implementing two strategies. On the one hand, diagrams already reported on the *NiAl* alloy were compiled; on the other, TTT diagrams were generated using the *Thermo-Calc™* software. A database of 96 TTT diagrams for the $\gamma^{\prime }$ phase with different chemical compositions was created, of which 66% were used for the training phase, and 33% for validation. The presented methodology analyzes the prediction of the nose point and the complete curve of the TTT diagrams for the binary *NiAl*.

It is concluded that the multilayer perceptron algorithm is the most efficient. A hybrid model was proposed to predict the complete TTT diagram. This model is formed by a perceptron-multilayer network configured with three hidden layers and another perceptron-multilayer network configured with one hidden layer to predict the nose point. In addition, a multilayer perceptron monolayer network was used to predict the top and bottom of the complete curve. The determination of the complete curve starts at the nose point.

The proposal presented allows the reduction by several hours of computation via *Thermo-Calc™* or long experimental days in calculating TTT diagrams. The proposed model does not require an end user with significant technical specialization, since only the chemical composition and temperature range to be analyzed must be entered and the system returns the results with an accuracy greater than 95%, on average. This precision was calculated along the upper and lower limits of the established chemical composition. As we approach these limits, the accuracy of the calculated diagram tends to decrease. This is because the limits in the diagrams go from nanoseconds to the bottom and hours to the top.

In future works, the prediction of the TTT diagrams will be optimized at the limits of the chemical composition and at the limits of the diagrams individually where there is a precipitation of the $\gamma^{\prime }$ phase. On the other hand, the use of new programming paradigms for the coding of neural networks, for example, parallel programming, will improve the performance of prediction.

## Figures and Tables

**Table 1:** Related works for TTT prediction. Due to the novelty of the use of ML techniques in the prediction of TTT diagrams, there are still few reported works.

Related Works	Prediction	Implemented Algorithm/Architecture	Material
[4]	TTT diagrams	Backpropagation multilayer	$Ti_{6}$-$Al_{4}$-V
[7]	$\beta_{tr}$ Temperature phase	Backpropagation Multilayer	Titanium alloys
[6]	TTT diagrams		Titanium alloys
[5]	TTT diagrams of ultrafine bainitic	Conjugate gradient algorithm	Commercial steels
[13]	CCT diagrams	Backpropagation multilayer	Steels
[12]	TTT diagrams	Nucleation theory	Metallic glasses based
[8]	TTT diagrams	Support vector machine	High carbon steels
[17]	Nucleation lag Time	Deep neural networks	Iron and steelmaking Slags
[9]	TTT diagrams	Backpropagation, random committee and random forest algorithms	Stainless steels
[11]	SH-CCT diagrams	Random forest, k-nearest and random committee algorithms	Ni-Cr-Mo Steels
[14]	Austenite decomposition during cooling	Long short-term memory	Medium carbon steel
[15]	TTT diagrams	Support vector machine	Pearlitic steel
[16]	Optimal design of hot-dip galvanized TT	Backpropagation multilayer and genetic algorithm	Galvanized steel

**Table 2:** Parameters used for the simulation of TTT diagrams in the software *Thermo-Calc™*.

Input	Parameters
Ni composition	Balance
Al composition	5.3, 5.4, …, 13.2, 13.3% wt
Elastic properties	Cubic
Min. temperature	323.15 °K
Max. temperature	1640.15 °K
Matrix phase	FCC (Gamma)
Precipitation phase	FCC L#12 (Gamma prime)
Grain size	100, 1000, 10,000 μm

**Table 3:** Experimental design to predict the nose point and the complete TTT curves with different inputs. Temperature (T) and time (t). Table 2 shows the parameter values.

DoE	Algorithms	Prediction	Strategy 1		Strategy 2	
			Input	Output	Input	Output
Exp. 1	SVM–MLP	nose point	Al %	t	Al %	T
Exp. 2	SVM–MLP		Al %	T	Al % and T	t
Exp. 3	MLP		Al %	t, T	-	-
Exp. 4	SVM–MLP– LSTM	complete curve	Al %, $\left[T_{max},T_{min}\right]$	t	-	-

**Table 4:** RMSE of the predicted data compared to *Thermo-Calc™*. The best option, with lower RMSE, was Exp. 1 (bold number), configured as MLP (three-layer) algorithm.

		RMSE		Score	
	Algorithm	Time (s)	Temperature (K)	Time (s)	Temperature (K)
Exp. 1	SVM	10,294.3788	44.9792	0.9921	0.9542
	MP1	1016.3671	3.4586	0.9999	0.9998
	MP2	4108.3471	4.6268	0.9999	0.9991
	MP3	**279.1034 **	6.2080	0.9999	0.9995
Exp. 2	SVM	11,181.6751	50.1539	0.9945	0.9542
	MP1	1207.1683	4.2720	0.9991	0.9998
	MP2	3987.9322	5.0901	0.9993	0.9991
	MP3	3553.9679	11.3034	0.9990	0.9993
Exp. 3	MP1	11,176.8126	**3.3762**	0.9997	
	MP2	11,182.3462	10.5387	0.9978	
	MP3	11,182.5408	11.7169	0.9999	

**Table 5:** Comparison of the different RMSE (in seconds) calculated for the different algorithms evaluated in Exp. 4. Figure 4 shows the predicted TTT curves.

Algorithm	6.1 Al	7.3 Al	9.7 Al	10.3 Al	10.9 Al	12.1 Al	Average
MLP1	**8515.7221**	**192.8856**	**11.5931**	**8.2832**	**2.2683**	13.6867	**1457.407**
MLP2	8681.1456	821.4856	27.1019	10.0785	23.1313	97.0063	1609.992
MLP3	8833.3507	8621.8408	43.7824	39.2825	38.3795	**10.8692**	2931.251
LSTM1	*Inf *	*Inf*	*Inf*	*Inf*	*Inf*	*Inf*	*Inf*
LSTM2	630,231.3461	8211.0746	99.2658	49.9114	23.9607	17.1395	106,438.8
SVM	65,192.8549	1198.1191	17.5913	44.3813	38.0452	10.8817	11,083.65

**Table 6:** Accuracy achieved in predicting the complete curve of **Exp. 4** using the Kolmogorov–Smirnov goodness-of-fit test. It is observed that the mono-layer MLP network is the one that obtains the best precision.

Algorithm	6.1 Al	7.3 Al	9.7 Al	10.3 Al	10.9 Al	12.1 Al	Average
MLP1	**81.1978**	**82.7202**	**89.2906**	**88.9285**	**95.0425**	76.7524	**85.6553**
MLP2	76.7708	77.031	63.9706	63.2793	60.1704	53.4611	65.7805
MLP3	42.6717	59.8222	55.9556	58.2392	58.2535	72.2924	57.8724
LSTM1	*Inf*	*Inf*	*Inf*	*Inf*	*Inf*	*Inf*	*Inf*
LSTM2	29.51	57.9536	60.8414	64.4185	74.3508	**81.3568**	61.4051
SVM	51.0803	44.8709	49.0361	52.861	54.5565	24.1036	46.0847

**Table 7:** Table showing the RMSE and accuracy (Acc.) calculated for the proposed hybrid model at different, randomly selected chemical compositions (see Figure 6). The general accuracy of our proposal is **96.59**.

	6.1 Al	7.3 Al	9.7 Al	10.3 Al	10.9 Al	12.1 Al
**RMSE**	43.89	32.37	21.63	21.39	26.74	51.86
**Acc.**	93.74	98.17	98.78	99.96	97.88	91.03

## Data Availability

The datasets generated during and/or analyzed during the current study are available from the corresponding author on reasonable request.
